# Reply to Chi et al.

**DOI:** 10.1016/j.jid.2016.08.026

**Published:** 2017-01

**Authors:** Grace Kaushal, Emanuel Rognoni, Beate M. Lichtenberger, Ryan R. Driskell, Kai Kretzschmar, Esther Hoste, Fiona M. Watt

**Affiliations:** 1 University College London Institute of Child Health, London, UK; 2 Centre for Stem Cells and Regenerative Medicine at King's College London, London, UK; 3 Department of Dermatology, Medical University of Vienna, Skin and Endothelium Research Division, Vienna, Austria; 4 Hubrecht Institute-Royal Netherlands Academy of Arts and Sciences (KNAW) and University Medical Centre (UMC) Utrecht, The Netherlands; 5 Unit for Cellular and Molecular (Patho)physiology, Inflammation Research Center, VIB, Ghent, Belgium and Department of Biomedical Molecular Biology, Ghent University, Ghent, Belgium

**Keywords:** DP, dermal papilla

To the Editor

We are grateful to Bruce Morgan and his colleagues for engaging in a dialog over our own article (Kaushal et al., 2015). We believe that open discussion about conflicting observations is important in moving forward research—far too often results that are at odds with one another are simply ignored.

There are certainly aspects of the Chi et al. (2017) study that are superior to ours. In particular, we agree that there are advantages of studying the dermal papilla (DP) in unpigmented skin, and we note that the quantitation by Chi et al. is based on a larger number of hair follicles, and mice, than in our study.

Chi et al. (2017) suggest that our results are due to misidentification of hair follicle types. We agree that DP size alone cannot be used to identify hair follicle type. We analyzed skin of P65 mice in which the hair follicle cycle was asynchronous so that we could report on anagen (stage IV; early anagen of the second hair follicle cycle, according to the classification of Müller-Röver et al. [2001]) and telogen follicles from the same mice. We believe that Chi et al. are looking at a later anagen stage. Given the low frequency of non-zigzag hairs in our samples, it is unlikely that they would skew our data.

The tissue in our article (Kaushal et al., 2015) was not flash frozen but fixed with 4% paraformaldehyde before being cryopreserved in Optimal Cutting Temperature medium. Tissue preservation under these conditions is excellent (Driskell et al., 2012, Driskell et al., 2013). The movies in the Supplemental Material of Kaushal et al. show that the 60-um–thick horizontal whole mounts are sufficient to capture all of the cells within a DP.

Several differences in the experimental approaches could contribute to the different results:

1. Chi et al. (2017) use a constitutively expressed Cre recombinase (Cre), not a tamoxifen inducible Cre (CreER), for genetic labeling and thus do not obtain temporal control of Cre activity. Further, no control for efficient recombination/β-catenin stabilization using this Cre line is provided in the current or previous (Enshell-Seijffers et al., 2010) study.
2. In the 2010 *Developmental Cell* article by Enshell-Seijffers et al., the researchers describe Corin-Cre activity as being first detected at P3 in some DP cells; however, Corin is not homogenously expressed in all DP cells until P7. In contrast, Kaushal et al. (2015) treated mice with tamoxifen to induce CreER at P1 and P2, before Corin-Cre is active. The use of different promoters to drive Cre expression and the activation of Cre at different times prevents direct comparison of the results.
3. Having worked with a number of different reporter lines, in our opinion the Rosa26-tdTomato reporter mouse line of Kaushal et al. (2015) is superior to the Rosa26-YFP reporter used by Chi et al. (2017), in terms of both recombination sensitivity and fluorophore expression level.

Finally, two recent articles by Zhou et al., 2016a, Zhou et al., 2016b support our conclusions (Kaushal et al., 2015). These researchers found that “expression of ΔN-β-catenin in CD133^+^ DP cells leads to increased DP cell proliferation” (p. 12) and that “in line with this finding, the number of DP cells was increased in mutant hair follicles. . . . Analysis of skin histology showed that the mean size of DPs in mutant CD133-CreERT2; Rosa-rtTA; tetO-Ctnnb1ΔN hair follicles . . . was increased compared with controls during early anagen stages” (p. 14) (Zhou et al., 2016a). These researchers have further reported that expression of a stabilized form of β-catenin promotes clonal growth of CD133^+^ DP cells in an in vitro three-dimensional hydrogel culture and on transplantation promoted in vivo hair growth in reconstituted skin compared with control cells (Zhou et al., 2016b).

In closing, we wish to thank you once again for the opportunity to discuss our results and look forward to further important contributions from the Morgan lab on the regulation and function of the dermal papilla.

## ORCIDs

Beate M. Lichtenberger: http://orcid.org/0000-0001-6882-0257

Kai Kretzschmar: http://orcid.org/0000-0002-4745-6684

Emanuel Rognoni: http://orcid.org/0000-0001-6050-2860

Fiona M. Watt: http://orcid.org/0000-0001-9151-5154

## Conflict of Interest

The authors state no conflict of interest.
